
JOURNAL INFORMATION
==============================
NLM Title Abbreviation: ScientificWorldJournal
Journal ID: ScientificWorldJournal
NLM Journal ID: 101131163
Journal ID: TSWJ

The Scientific World Journal

ISSN: 2356-6140
EISSN: 1537-744X
Publisher: Wiley

ARTICLE INFORMATION
==============================
PMCID: PMC5974853
DOI: 10.1100/tsw.2003.86
Article ID: 710985
Article version: 1
Subjects: Announcement

Announcement—Ecological Modeling, a Distance Learning Course

Jørgensen Sven *
Institute A, Section Environmental Chemistry, University Park 2, 2100 Copenhagen Ǿ, Denmark
*Sven Jørgensen: sej@dfh.dk
Publication date: 2003
Electronic publication date: 2003 Sep 3
Volume: 3
First page: 867
Last page: 869
Copyright: Copyright © 2003 Sven Jørgensen.
Copyright year: 2003
License: This is an open access article distributed under the Creative Commons Attribution License, which permits unrestricted use, distribution, and reproduction in any medium, provided the original work is properly cited.
License URL: https://creativecommons.org/licenses/by/3.0/

Keywords: distance learning, environmental models, parameters in ecotoxicology, ecotoxicology models, biogeochemical models, food chain models, eutrophication models, energy circuit language, ecosystems and communities, environmental toxicology, population dynamics models

==============================
Announcement

TheScientificWorldJOURNAL (2003) 3, 867–869
ISSN 1537-744X; DOI 10.1100/tsw.2003.

### Announcement — *Ecological Modeling*, a Distance Learning Course

Sven Erik Jørgensen

Institute A, Section Environmental Chemistry, University Park 2, 2100 Copenhagen Ø, Denmark; Fax: (45) 35306001

Email: sej@dfh.dk

*Published September 3, 2003*

---

**KEY WORDS:** distance learning, environmental models, parameters in ecotoxicology, ecotoxicology models, biogeochemical models, food chain models, eutrophication models, energy circuit language, ecosystems and communities, environmental toxicology, population dynamics models

**DOMAINS:** ecosystems and communities, terrestrial environmental toxicology, environmental chemistry, environmental modeling

---

An internet-based distance-learning course on Ecological Modeling[1] is available to provide an introduction to environmental science modeling. Broadly, the topics will cover:

- Modeling as a Tool in Environmental Management and Research
- Eutrophication and Food Chain Models
- Ecotoxicological Models

The course is suitable for all those with an interest in gaining a sound knowledge of modeling environmental, ecotoxicological, and ecological data, including researchers within academic institutions and industry, environmental consultants, and environmental managers around the world.

The course was launched in April 2001, and students can join the course at any date. Course participants can work at their own pace and are assisted by the Course Tutor, S.E. Jørgensen of the University of Copenhagen, Denmark.

The course will consist of eight steps corresponding to chapters in the textbook *Fundamentals of Ecological Modeling*[2]. A third edition of the book was published August 2001. In addition to the exercises and problems within the textbook chapters, each step will consist of additional exercises and a stepwise development of participants' own models. The results of exercises and the model development will be reviewed and commented by the Course Tutor. The eight steps and corresponding chapters with *Fundamentals of Ecological Modeling* (3rd ed.) are:

*Corresponding author.
©2003 with author.

1. Chapters 1 + 2. *Introduction and How to go about Modeling.* The database, the background problem and the conceptual diagram of the participant's own model is discussed.

2. Chapter 3. *Sub-Models and Process Descriptions*. The equations for the processes is participant's own model are presented and discussed.

3. Chapter 4. *Conceptual Models.* The conceptual diagram is discussed again after the processes have been expressed by equations.

4. Chapter 5. *Steady State Models*. The steady state edition of participant's own model is discussed, possibly by use of ECOPATH[3].

5. Chapter 6. *Population Dynamic Models.* It is presumed that the participant's own model is a dynamic biogeochemical model, but there will be occasion to use a software based on H.T. Odum's energy circuit language applied to a food chain[4]. The software is self-instructive and facilitates self-testing.

6. Chapter 7. *Biogeochemical Models*. The participant's own model is calibrated and validated. Three software testing eutrophication models can be used for model training.

7. Chapter 8. *Ecotoxicology Models*. Software denoted WINTOX[5] is used to estimate parameters for ecotoxocological parameters. Five additional exercises in the use of the software are applied.

8. Chapter 9. Recent Development in Ecological Modeling. Final discussions on model results, including discussion on the suitability of one of the new approaches presented in Chapter 9 used for problem solving.

A course fee applies which includes two textbooks (F*undamentals of Ecological Modelling* and *Principles of Pollution Abatement*), five software packages (three eutrophication models, a self-instructive software based on H.T. Odum's Energy circuit language applied to food chains', and WINTOX applied to the estimation of ecotoxicological parameters). Textbook and software will be sent at the beginning of course. The course can also provide STELLA[6] software, which is recommended for all small and medium size models, using small and medium-sized databases. STELLA is used extensively in the textbook to give illustrative modeling examples. Participants are also offered discounts on modeling books published by Lewis Publishers, U.S.

All interested in joining the course should send an e-mail to Professor Dr. Eng. Dr. Scient. S.E. Jørgensen at sej@dfh.dk. See also the course web site at http://www.ecologicalmodel.net/.

### REFERENCES

1. Internet course on Ecological Modelling, 2001: URL: www.enviromod.subnet.dk/.
2. Jørgensen, S.E. (2001) Fundamentals of Ecological Modeling. Elsevier Science.
3. ECOPATH 2000. Software. Published by Villy Christensen and David Pauly, Vancouver. URL: www.enviromod.subnet.dk.

4. Odum, H.T. Software, 1988 Software based on “Energy circuit language applied to food chains’ and published by Cathy Ewells.
5. WINTOX 1997, Software published by S.E. Jørgensen, Bent Halling Sørensen og Henrik Mahler
6. STELLA 2000. Version 5.1. Software published by High Performance LTD.

**This article should be referenced as follows:**

Sven Erik Jørgensen. (2003) Announcement — Ecological Modeling, a Distance Learning Course. *TheScientificWorldJOURNAL* **3**, 867–869.
